# Assessment of Passive Upper Limb Stiffness and Its Function in Post-Stroke Individuals Wearing an Inertial Sensor during the Pendulum Test

**DOI:** 10.3390/s23073487

**Published:** 2023-03-27

**Authors:** Milene Soares Nogueira de Lima, Clarissa Cardoso dos Santos Couto Paz, Thais Gontijo Ribeiro, Emerson Fachin-Martins

**Affiliations:** 1 Program in Health Sciences and Technologies, Faculdade de Ceilândia, Universidade de Brasília, Brasília 70910-900, Brazil; 2 Course of Physiotherapy, Faculdade de Ceilândia, Universidade de Brasília, Brasília 70719-080, Brazil; clarissacardososcp@gmail.com (C.C.d.S.C.P.);; 3 Secretaria de Saúde do Distrito Federal, Brasília 72445-020, Brazil

**Keywords:** spasticity, stroke, stiffness, the pendulum test, inertial sensor, upper limb

## Abstract

This article proposes the evaluation of the passive movement of the affected elbow during the pendulum test in people with stroke and its correlation with the main clinical scales (Modified Ashworth Scale, Motor Activity Log, and Fulg Meyer). An inertial sensor was attached to the forearm of seven subjects, who then passively flexed and extended the elbow. Joint angles and variables that indicate viscoelastic properties, stiffness (K), damping (B), E1 amp, F1 amp, and relaxation indices were collected. The results show that the FM scale is significantly correlated with the natural frequency (*p* = 0.024). The MAL amount-of-use score correlates with the natural frequency (*p* = 0.024). The variables E1 amp, F1 amp, RI, and ERI are not correlated with the clinical scales, but they correlate with each other; the variable E1 amp correlates with F1 amp (*p* = 0.024) and RI (*p* = 0.024), while F1 amp correlates with ERI (*p* = 0.024). There was also a correlation between the natural frequency and K (r = 0.96, *p* = 0.003). Non-linear results were found for the properties of the elbow joint during the pendulum test, which may be due to the presence of neural and non-neural factors. These results may serve as a reference for future studies if alternative scales do not provide an accurate reflection.

## 1. Introduction

Neural function disorders caused by stroke are responsible for cognitive, motor, and sensory dysfunctions and can result in clinical conditions of varying disabilities. Consequently, post-stroke motor impairments related to neuromusculoskeletal movement are characterized by muscle weakness and both reduced muscle activation and coactivation, which decrease one’s ability to perform efficient movements [1]. In the chronic phase of stroke (after 6 months), between 55 and 75% of individuals present significant and permanent functional impairments following a period of spontaneous recovery, with less severe impairments influenced by factors such as age, location, and the severity of the impairment [2]. The life of post-stroke patients is challenging, considering the series of situations to which the individual needs to adapt, such as work, interpersonal relationships, finances, and dependence on support networks, thus impacting motor recovery [3]. The functional relevance of the shoulder and elbow for activities of daily living is well described in the literature. In addition, muscle weakness that compromises motor performance is related to reduced speed, a greater tendency toward fatigue, reduced peak torque, and muscle inactivation [4].

The interruption and inhibition of spinal reflexes add obstacles to the performance of movements, generating muscle hyperactivity, which is related to an increase in muscle tone, known as hypertonia. This occurs when the muscle loses its ability to stretch or perform a sequence of rapid muscle contractions [5]. These mechanisms of hypertonia are related to the stretch reflex, known more specifically as spasticity. Hypertonia, more broadly, is related to passive displacements and is not influenced by the speed of movement, which, because of muscle contracture, is sometimes classified as non-reflex hypertonia or intrinsic hypertonia [6].

As part of evaluations performed in clinical routines, clinicians assess the issue of muscle stiffness through passive stretching in subjects with stroke. At different speeds, characteristics such as muscle contractures and increased stiffness are not able to be completely distinguished and may provide results that do not correspond to functionality [7,8]. The Modified Ashworth Scale (MAS) is the most commonly used scale in clinical practice for measuring spasticity, the main clinical condition of stroke. The MAS is performed by a clinician manually moving the affected extremity of the stroke victim [9,10]. This scale presents reduced validity and reliability measures due to the lack of information on factors that influence spasticity; considering movement as the total sum of activity and non-neural mechanical characteristics, studies show that the scale is insufficient to assess spasticity [11].

In regular clinical practice, performance tests may not match the performance of patients outside of test situations. These behavioral measures focus primarily on functional independence as opposed to the quality and extent to which the affected limb is being used [9].

A valid and reliable method is needed to objectively measure and monitor the evolution of a stroke patient’s daily activities. In this context, a prospective method could be found in the use of inertial measurement units combined with the pendulum test. The technology of inertial measurement units is accessible and portable, providing an increasingly popular assessment method outside laboratory contexts [12]. Inertial sensors, devices that use accelerometers and gyroscopes to measure changes in motion and orientation, play an important role in the objective assessment of muscle tone and its alterations, such as spasticity, yet there is currently a lack of studies that corroborate the clinical scales in use and the measures of the combined results [13]. Inertial sensors are valuable, as they provide values for monitoring and evaluating individuals during neurorehabilitation, resulting in more objective data on physical progress and on how the upper limbs move in space [12,14,15]. Advanced linear and complex models have the capability to track varying trajectories over time, resulting in a diverse range of physiological outcomes [11,13,16]. 

The pendulum test measures the amount of swing or oscillation that occurs in the limb, which provides information about the individual’s muscle tone and stiffness. This test aids in the assessment of muscle tone during human performance, as there are skills and movement characteristics that change over time and reflect the complexity of the movement system through varying degrees of freedom of movement [10,14,17]. Many daily activities require the use of the elbow, which plays a crucial role in guiding and positioning the hand in space. The operating range of the elbow is typically between −30° of extension and 130° of flexion [18]. To understand muscle performance, we can compare its properties to that of a spring, whose stiffness potential and muscle tension determine its elasticity. Thus, specific calculations can demonstrate these parameters and their functionality [19].

In the current study, we used the pendulum test on the elbow joint and formulated parameters and essential quantification scales. Through research, therapists can gain insight into specific functional muscle tone dynamics following stroke, making it possible to offer patients personalized rehabilitation therapies. Thus, in the current article, we demonstrate the potential for using the pendulum test combined with an inertial sensor for assessing upper limb function. In addition to explaining the structure of the pendulum test and its correlations with the Fugl Meyer (FM) assessment scale, the Motor Activity Log (MAL), and the MAS, we cover subject-specific muscle tone dynamics.

## 2. Materials and Methods

### 2.1. Participants

The Human Research Ethics Committee approved the project’s developmental data as part of a prospective longitudinal study at the University of Brasília, Brazil. Participants were recruited from a stroke referral hospital. The inclusion criteria were individuals of both sexes, aged over 18 years, with an episode of stroke classified as chronic; a middle cerebral artery lesion identified via imaging (Computed Tomography or MRI) and confirmed by the medical team. The exclusion criteria were individuals using antidepressants, antipsychotics, or benzodiazepines; advanced systemic disease; the previous presence of any limiting pathology in the evaluated upper limb; intracranial hypertension or risk of clinical evolution; the presence of a pacemaker or cardiac stent or any intracranial metallic implant; prior brain surgery intervention; and severe, sensitive, or mixed motor aphasia. 

Three individuals with Parkinson’s disease, ankylosing spondylitis, and Alzheimer’s disease were excluded. Seven individuals who had previously had a stroke and were in the chronic phase met the criteria and volunteered for this investigation, providing their demographic information and written consent. The reported results were obtained from these seven participants. The demographic information of the patients is summarized in Table 1.

### 2.2. Clinical Assessments

The MAS, an assessment tool that is frequently used to measure the severity of spasticity, was utilized to assess the compromised upper limb in the current study. The MAS is a scale with a score that varies between 0 and 4, where 0 represents no increase in muscle tone. The scale demonstrates gradual increases until reaching the maximum evaluation score of 4, representing joint stiffness [16]. Additionally, the participants were assessed by FM, which is a commonly used and recommended clinical scale to evaluate sensorimotor impairments in subjects with stroke. This scale contains a possible total of 100 points for normal motor function, where the maximum score for the upper extremity is 66 and for the lower extremity is 34. For upper limbs, 0–15 was considered severe motor dysfunction, 16–34 was severe to moderate motor dysfunction, 35–53 was moderate to mild motor dysfunction, and 54–66 was mild motor dysfunction [20].

The MAL assessment instrument is considered to provide more comprehensive information, given that it is used outside the therapeutic environment. The instrument provides information on how much and how well the affected arm is used in 30 daily living activities, as well as provides a continuous picture of the quality of arm function [21]. The quantitative domain involves scores ranging from zero (“does not use the most affected upper limb”) to five (“uses the most affected upper limb the same way as before the stroke”). In the qualitative domain, the variation in scores ranges from zero (“the most affected upper limb is not used at all for the activity”) to five (“ability to use the most affected upper limb is as good as it was before the stroke”) [22]. The total score of the scale is calculated by summing the averages for each of the subscale categories. Before the pendulum test, all scales were applied by a trained team, following standardized procedures.

### 2.3. Experimental Setup

**Figure 1:**
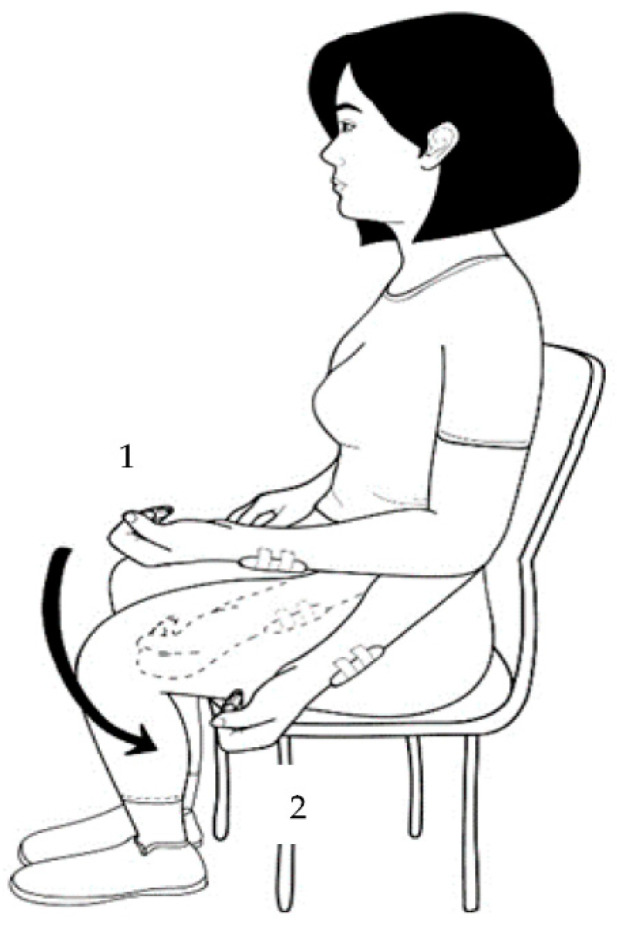
Location of the inertial sensor. The sensor was attached to the lateral side of the forearm: (1) starting position and (2) ending position.

The pendulum test was performed in a sitting position, with the elbow at 90° flexion, the shoulder at 0° flexion, and the forearm in supination. A sensor was placed on the forearm close to the wrist, and the examiner positioned the participant’s limb as described above. During the test, the participant was asked to relax the upper limb, as the test depends on the limb being relaxed. The same evaluator performed all evaluations, starting with the elbow being held stationary for calibration for a period of 3 s in the initial pose. The inherent viscoelastic properties of the joint and surrounding tissues, along with the mass of the arm and elbow motion, will eventually cause the arm to approach a vertical position [23]. In cases of spasticity, the affected upper limb loses normal oscillation and reverses the direction of movement (Figure 1).

Each subject completed two successful attempts for each condition, with a time interval of 5 min between trials. Trials were successful when the participant allowed their arm to swing freely, did not actively swing their arm, and did not produce movements, such as moving the shoulder forward (Figure 1).

### 2.4. Data Collection

In this study, we used an off-the-shelf wearable device that incorporated a three-axis accelerometer and a three-axis magnetometer (G-WALK, Sensor, BTS bioengineering, Milan, Italy). The device with the inertial sensor was on the wrist of the affected upper limb during the pendulum test and collected the kinematic parameters of movement and its impact on the evaluated structure. BTS G-Studio software was used to process the data. The pendulum test was performed to measure the level of spasticity and muscle tone of the affected limb of the individual [24].

### 2.5. Feature Extraction

The affected upper limb was classified according to spasticity severity and non-use. After this pre-processing phase, these characteristics were computed. Two sets of features were prepared to investigate the impacts of feature types on classification performance and pendulum testing. The most common statistical features—for example, the median, standard deviation, and correlation—were extracted from the data sets. In addition, resources were computed from the kinematic measurements of each pendulum test by using the following variables: initial angle, corresponding to the elbow in a neutral position; final angle, corresponding to the angle of repose at the end of the test with the upper limb in extension; the angle of the first extension peak (E1); the angle of the first flexion peak (F1), with an initial extension range (E1Amp = F1 − E1) and an initial flexion range (F1Amp = F1-initial angle); plateau amplitude (PA = measure between the final angle and the initial angle); the relaxation index (RI = F1Amp/PA) and extension relaxation index (ERI = E1Amp/PA), which represent the angle of the first normalized peak of extension and the angle of the first normalized peak of flexion, respectively; and the duration from the start angle to the end angle (D), according to the guidelines of Valle et al. [25]. We present a list of the variables we used to obtain our outcome measures in Table 2. The variables related to the measurement of stiffness were obtained from the following equations.

The ratio of stiffness to mass provides the natural frequency (*ω*), where T is the period of one cycle [26]:

ω = 2π/T(1)

With data from the first and second reversion angles at the end of flexion (F1 and F2), we were able to calculate the damping coefficient (B), stiffness coefficient (K), and damping ratio (ζ) variables as follows [27]:

(2) $$\zeta =\sqrt{\frac{{\left(\ln D\right)}^{2}}{4\pi^{2}+{\left(\ln D\right)}^{2}}}$$

The cycle peak angle for the next peak angle in the next cycle is the value of *D*:

B = 2.ζ.ω.*I* (3)

The moment of inertia is given through the axis of rotation of the elbow.

(4) $$K=I.\omega^{2}$$

*I* and the mass characteristics (elbow and forearm segment) were calculated for each subject according to Winter [26]. For example, for Subject 1,

T = 3.16 − 2.14 = 1.02

ω = 2π/T = 2.3.14/1.02 = 6.15 *

Thus, we have ω.

For

lnD = 1.65

we have

$$\zeta =\sqrt{\frac{{\left(\ln D\right)}^{2}}{4\pi^{2}+{\left(\ln D\right)}^{2}}}=\sqrt{\frac{{\left(1.659813084\right)}^{2}}{4.3.14^{2}+{\left(1.65\right)}^{2}}}=0.25\;*$$

which is ζ.

For *I*, we can obtain

$$I=arm\;mass.{\left(distance\;of\;the\;center\;of\;mass\;to\;elbow\right)}^{2}$$

$$I=arm\;mass.{\left(arm\;length\times 0.318\right)}^{2}$$

$$I=1.54{\left(0.245\times 0.318\right)}^{2}=0.009\;*$$

and thus,

B=2.(0.25). (6.1). (53.1)=0.029* 

Finally,

$$K=0.009\times {\left(6.15\right)}^{2}=0.355\;*$$

* Approximate values.

### 2.6. Statistical Analysis

Descriptive statistics were used to summarize the collected data. Quantitative data are presented as the median and standard deviation. Spearman’s correlation was used to analyze the associations between the variables. The level of significance was set at *p* < 0.05. All analyses were performed using Graph Pad Prism version 8.4.1 for Windows (Graph Pad Software, San Diego, CA, USA). 

## 3. Results

### 3.1. Characteristics

Seven people after chronic stroke participated in this study (five females and two males), with a mean age of 51.85 ± 11.18 years (ranging from 43 to 72 years), a mean weight of 63.42 ± 6.27 kg (ranging from 54 to 73 kg), and a mean height of 1.60 ± 0.11 m (ranging from 1.40 to 1.77 m).

### 3.2. Variables Related to the Scales

In the assessment of elbow spasticity using the MAS, two individuals received a score of 1, three received a score of 2, and two received a score of 0. The median score was 2.0 ± 0.54. One participant was classified with mild motor dysfunction, four individuals had moderate to mild motor dysfunction, and two participants had severe dysfunction according to the classification of the FM scale, and the median of the scale was 35± 14.02. Regarding the use of the upper limb on the MAL, the data showed that the median of the MAL amount-of-limb-use and MAL quality-of-use scores were 2.0 ± 0.91 and 1.0± 0.60, respectively. The information is summarized in Table 3.

### 3.3. Variables Related to the Inertial Sensor and Pendulum Test

The results of assessing extension and flexion movements by the pendulum test according to segmentation techniques are reported in Table 4.

### 3.4. Graphs Related to Participants’ Tests during the Pendulum Test

**Figure 2:**
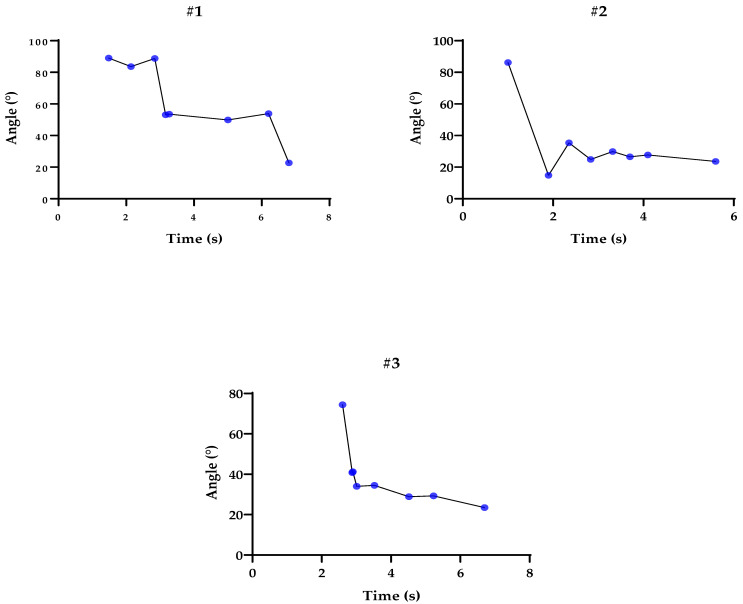
Representation of the pendulum tests using the inertial sensor: Flexion and extension peaks represented by the blue dots, according to the trajectory of angles (°) and time (s) for participants #1, #2, and #3.

**Figure 3:**
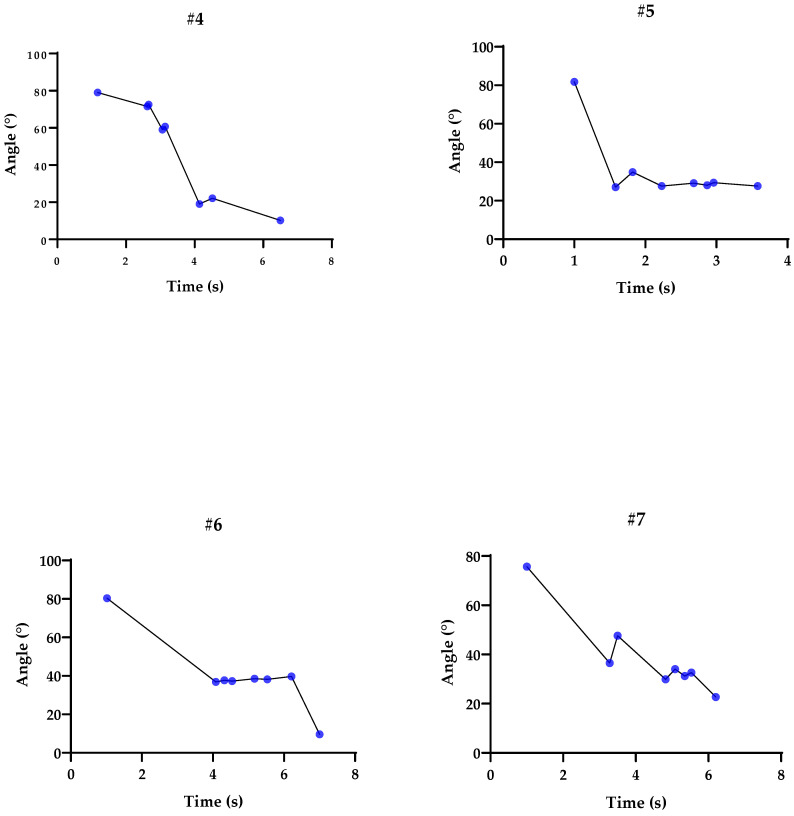
Representation of the pendulum tests using the inertial sensor: Flexion and extension peaks represented by the blue dots, according to the trajectory of angles (°) and time (s) for participants #4, #5, #6, and #7.

To analyze and validate our statistics, we used individual graphs of each participant. Figure 2 and Figure 3 depict the elbow angles, and the blue dots represent peak flexion and extension. Each interval lasted between 4 and 8 s, including a 3 s stationary calibration period in the initial pose. The subjects performed the movements at a normal speed, as used in daily life, keeping the rest of the body still and trying not to compensate with other structures, such as the shoulder.

### 3.5. Correlations

**Figure 4:**
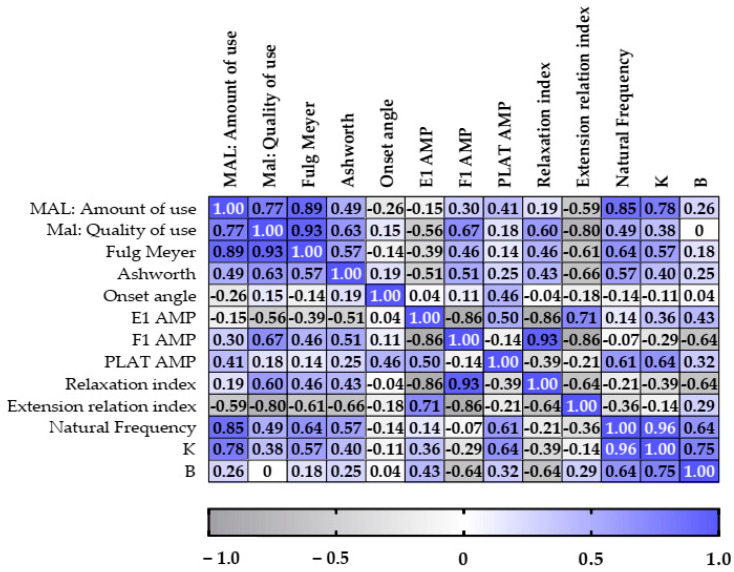
Correlation data of the clinical scales and the variables evaluated in the pendulum test with the inertial sensor according to Spearman’s correlation. The results are represented by color value gradients (the darker the color, the greater the correlation).

The Fugl Meyer scale showed significant correlations with the MAL amount-of-use score (r = 0.89, *p* = 0.01) and the MAL quality-of-use score (r = 0.93, *p* = 0.008). The MAL amount-of-use score correlates with the MAL quality-of-use score (r = 0.93, *p* = 0.05) and with the natural frequency (r = 0.85, *p* = 0.024). The variable E1 amp correlates with F1 AMP (r = −0.86, *P* = 0.024) and RI (r = −0.86, *p* = 0.024), while F1 amp correlates with ERI (r = −0.86, *p* = 0.024). There was also a correlation between the natural frequency and K (r = 0.96, *p* = 0.003). There were no other correlations that could be considered significant, as shown in Figure 4.

## 4. Discussion

The current study aimed to determine whether passive elbow movement in the pendulum test combined with data from an inertial sensor correlates with elements of a series of objective measures. This approach was motivated by the fact that the inertial sensor is a small, light, and practical device, which does not cause discomfort to the subjects. The sensor provides qualitative and quantitative information related to muscle tone [28,29,30,31], such as the regularity of movement balance, observation of the sinusoidal curve with irregularities, and more frequent alterations in individuals with spasticity.

The feasibility of using sensor technology with the pendulum test to assess spasticity in individuals is significant. Bohannon et al. demonstrated the validity of the pendulum test with correlations ≥ 0.57 in their measurements and concluded that the pendulum test effectively detects the presence of spasticity [19]. Additionally, White et al. demonstrated the high reliability of the test–retest variables, suggesting that the pendulum test provides an objective and reliable method for assessing spasticity [20]. The study by Gohary et al. evaluated kinematic models designed to control robotic arms with state-space models for continuously estimating human shoulder and elbow angles using two wearable inertial measurement units. The mean correlation coefficient in this study for all movement tasks across all subjects was r ≥ 0.95. It is thus suggested that the pendulum test presents reliability, repeatability, and validity [21].

The properties of the elbow joint are complex, and, to date, assessment methods have demonstrated significant limitations. For example, most scales are based on the rater’s interpretation of items and predetermined ordinal values; therefore, the reliability and objectivity of these scales are questionable. Considering that spasticity is generally easy to recognize, yet not so easy to quantify, the perception of the improvement or worsening of muscle tone may not be conceived or may contain errors [32].

In addition to neural aspects, changes in tissue properties lead to changes in movement dynamics; physiologically, skeletal muscle is subjected to a wide variety of tensions, often functioning with varying degrees of relaxation and stress. These changes in movement dynamics are caused by more than neural aspects, such as changes in tissue properties; physiologically, skeletal muscle is subjected to a wide variety of stresses and strain rates. Thus, muscle fibers have a complex viscosity, which is dependent on the tension and rate of tension [33]. These tissue phenomena usually result from reversible changes in the microstructure. In individuals with stroke, rigidity makes it difficult to initiate movement after a period of immobility due to the reduction in the number of actin–myosin cross-bridges [34]. When the muscle subsequently becomes immobile, the attachments reform, and the muscle becomes rigid again, which generates motor behaviors that are difficult to predict and that inhibit voluntary movement [35].

We interpreted muscle tone measurements and their correlations with clinical measurement scales. The correlations between pendulum variables, the FM scale, and the MAS scale were not significant. A possible explanation may be demonstrated in the recent study by Daily et al. [36], who observed that during the application of the FM scale in patients with stroke, despite the presentation of satisfactory muscle strength, additional hours of motor practice are necessary for individuals to obtain control of joint movement in isolation, and this is performed with synergy and without abnormal co-contractions. Thus, difficulty in muscle coordination was observed in our experiment while performing the test, which may not be due to a lack of strength but due to a lack of coordination. 

Another trend was shown in the study by Pudink et al., who demonstrate that variations in arm joint angles are altered by stiffness and damping. The relationship that occurs is that the inequalities between agonists, which have high muscle tone, may lead to insufficient activation, and the stiffness tends to increase in the shoulder–hand direction and decrease in the orthogonal direction as the elbow is extended [37]. When applying the FM scale, different classifications were observed regarding severity. Our results showed heterogeneity even with individuals in the chronic phase, which may be attributed to factors such as access to neurorehabilitation programs, the severity of the stroke, and the possibility of finding several pathways for motor recovery [38]. The MAS only offers qualitative information, which requires reproducibility. The lack of correlation in all variables seems to resemble the study by Huang et al. on differences in the degree of hypertonia, bearing in mind that, in our sample, two individuals obtained a score of 0 on the MAS, indicating no increase in muscle tone, while the other five participants showed an increase in tone [39]. Although the FM scale is not related to viscoelastic properties, the correlation was significant with the MAS (r = 0.89, *p* = 0.012). Thus, the values found in our study corroborate the literature, as well as correlate with the FM scale, in that using the less-affected limb tends to predict low functionality performance [22,36].

This is the first study to correlate the amount of use of the affected upper limb, assessed by the MAL scale, with the viscoelasticity scales; however, only the natural frequency seems to be significant (r = 0.85, *p* = 0.024). Our natural frequency results corroborate previous studies [40] in which joint stiffness increased as the movement frequency increased, and it seems that, for this reason, there was also a correlation between the natural frequency and K (r = 0.96, *p* = 0.003).

The E1 amp and F1 amp parameters had lower correlations with the index that represents stiffness (K), and the value of F1 amp presented a lower correlation with the extension relaxation index (ERI). One possible inference from this result is that the capacity to resist the movement of joint tissues during movement depends on the frictional force, that is, on the viscosity of these tissues, stiffness increases in the distal region, and, perhaps, the muscles have the greatest participation in the resistance produced. 

Figure 2 and Figure 3 present graphs of the range of motion in the pendulum test, demonstrating oscillations in the flexion and extension of the elbow. It is important to note that muscle activity was not evaluated, which would be fundamental to better mapping differences in activation patterns between agonists and antagonists. In addition, future studies may complement the behavior of muscle strength during the test [41]. It was observed that the affected upper limbs have difficulty starting the test in the appropriate range (~90°), and that the decline performed cannot reach the end of the range of motion. According to the study carried out by Lin et al. [24], constant stiffness values that were estimated in healthy subjects in previous studies indicate that the 2.62 Nm/rad for men and 2.26 Nm/rad for women in our study are below the published data in all cases, and for damping, our values of 0.40 Nm/rad for men and 0.603 Nm/rad for women are lower than the values presented in other studies [27]. Finally, near the end of the test, the trajectories did not reach the steady state. One possible explanation for this phenomenon is that reflex or voluntary muscle contractions are possible sources of inaccuracy, just as stiffness has passive components related to tissues and is modulated through neural control, considering that factors such as biomechanical distortions cause a quantifiable increase in stiffness, just as there is the consideration that tonus is a factor of spasticity where elements such as contractures, joint amplitude, and muscle spasms can manifest [40]. 

Ultimately, our work introduces a new quantitative assessment of upper limb function, based on the pendulum test during neurorehabilitation, for individuals following a stroke. As we collected movement data from only seven subjects and at only two time points, the generalizable evaluation criterion cannot be established. However, this model can serve as a method to predict alternative scales that are not an accurate reflection of the conditions of the viscoelastic properties of the individuals. This will allow us to carry out more experiments in the future.

## 5. Conclusions

This work not only successfully demonstrated how sensor technology with clinical variables, allied to the simple pendulum test, can be used to generate a clinically meaningful index in the future but also proposed a method that offers rehabilitation patients the opportunity to monitor stiffness. We used inertial data to assess the severity of elbow tone using the pendulum test. The parameters chosen in this work demonstrate stiffness and correlate with the FM, Motor Activity Log, and MAS scales. The data obtained were segmented using temporal and spatial processing methods. Due to the lack of standard measures that can be compared between individuals, the pendulum test and processing presented could be used as a way to compare affected and unaffected limbs.

## Figures and Tables

**Table 1:** Demographic information of the study participants.

Characteristics	#1	#2	#3	#4	#5	#6	#7
Sex	M	M	F	F	F	F	F
Age (years)	54	45	49	60	72	43	40
Height (m)	168	177	167	160	140	153	159
Weight (kg)	70	72	54	57	64	54	73
Time since stroke (months/days)	12.6	6.4	7.2	12.4	5.4	12.6	6.3
Stroke location (right/left)	L	R	L	L	L	R	L
Dominant side (right/left)	R	L	R	R	R	R	R

Legend: M—male; F—female; L—left; R—right.

**Table 2:** Selection of parameters considered for analysis with the inertial sensor.

List of Parameters (Measures)	
E1	First extension peak (*◦*/s)
F1	First flexion peak (*◦*/s)
E1Amp	F1 − E1 (*◦*)
F1Amp	F1-initial angle (*◦*)
PA	Measure between the final angle and the initial angle (*◦*)
RI	F1 Amp/PA (*◦*)/relaxation index
ERI	E1 Amp/PA (*◦*)/extension relaxation index
D	Duration from the start angle to the end angle (*◦*)
ω	Natural frequency
B	Damping coefficient
K	Stiffness coefficient
ζ	Damping ratio

**Table 3:** Results: the median and standard deviation of the clinical scales applied to individuals (*n* = 7) with stroke.

Scales	Subjects								
	#1	#2	#3	#4	#5	#6	#7	Median	SD
MAS	-	2	-	2	2	1	1	2.0	0.54
FM	14	34	18	35	56	39	38	35	14.02
Motor Activity Log—amount of limb use	0.0	1.5	0.5	2.0	2.0	2.5	2.0	2	0.91
Motor Activity Log—quality of use	0.0	1.0	0.0	0.5	1.5	1.3	1.0	1.0	0.60

**Table 4:** Summary of the results concerning the onset angle, the amplitude of extension (E1 amp) and amplitude of flexion (F1 amp), the relaxation index (RI), and the extension relaxation index (ERI) during the pendulum test.

Variable	Subjects								
	#1	#2	#3	#4	#5	#6	#7	Median	SD
Onset angle (°)	89	86.2	74.4	79	81.8	80.4	75.7	80.40	5.28
E1 amp	172.6	101	115.2	150.5	108.8	117.3	112.2	115.20	26.05
F1 amp	0.2	50.8	33.2	6.5	46.9	42.7	28.1	33.20	19.72
Plat Amp	66.3	62.6	50.9	68.8	54.2	70.8	53	62.60	8.16
RI	0.003	0.812	0.652	0.094	0.865	0.60	0.53	0.60	0.33
ERI	2.60	1.61	2.26	2.18	2.00	1.65	2.11	2.12	0.34
Natural frequency (rad/seg)	6.15	6.75	1.45	14.27	9.6	13.65	11.62	9.66	4.59
K (Nm/rad)	0.355	0.448	0.013	1.78	0.61	1.10	0.79	0.67	0.58
B (Nm.s.rad)	0.029	0.025	0.003	0.047	0.024	0.025	0.030	0.03	0.013

Legend: RI—relaxation index; ERI—extension relaxation index; K—stiffness coefficient; B—damping coefficient.

## Data Availability

Not applicable.
